# Prevalence of porcine cysticercosis in the Lake Kyoga Basin, Uganda

**DOI:** 10.1186/s12917-014-0239-y

**Published:** 2014-10-05

**Authors:** Zachary Nsadha, Lian F Thomas, Eric M Fèvre, George Nasinyama, Lonzy Ojok, Charles Waiswa

**Affiliations:** School of Veterinary Medicine and Animal Resources, Makerere University, P.O Box 7062, Kampala, Uganda; Centre for Immunity, Infection and Evolution, Institute for Immunology and Infection Research, School of Biological Sciences, University of Edinburgh, West Mains rd, Edinburgh, EH9 3JT UK; International Livestock Research Institute, P.O Box 30709, Nairobi, 00100 Kenya; Institute of Infection and Global Health, University of Liverpool, Leahurst Campus, Neston, CH64 7TE UK; Coordinating Office for Control of Trypanosomiasis in Uganda, P.O Box 16345, Wandegeya, Uganda

**Keywords:** *Taenia solium*, Cysticercosis, Public health, Zoonoses, Helminths, Neurocysticercosis, Prevalence, East Africa, Lake Kyoga Basin

## Abstract

**Background:**

*Taenia solium* is a zoonotic helminth with the potential to cause life threatening epilepsy in people through the aberrant larval infection of the brain called Neurocysticercosis (NCC). The pig is the intermediate host for *T. solium* where the larval form, cysticercus cellulosae, normally develops after the pig eats eggs of the parasite. Humans are the definitive host where the adult tapeworm develops and are infected through the consumption of poorly cooked, infected meat. *T. solium* has been acknowledged by the World Health Organization (WHO), Food and Agriculture Organization (FAO) and UK Department for International Development (DFID) as being a neglected zoonotic disease, and was recently included in the WHO roadmap for control of neglected tropical diseases. This neglect encompasses a lack of epidemiological data and a lack of validated, effective control strategies being adopted. Understanding the epidemiology of this parasite in the intermediate host is the first step towards designing suitable intervention strategies for the improvement of public health. This study was undertaken to provide an accurate and up-to-date estimate for the prevalence of porcine cysticercosis in the Lake Kyoga basin.

**Results:**

Sera from 378 pigs were analysed with the HP10 Antigen Enzyme Linked Immunosorbant Assay (ELISA) and the prevalence was found to be 25.7% (95% confidence interval 21.0% to 30.0%). Previous sero- surveillance in this region, using the B158/B60 Ag Elisa had indicated a prevalence of 8.6% in 2005 indicating a dramatic increase in prevalence (J. Parasitol Res, Article ID 375493, 2009) within a 6 year period.

**Conclusion:**

This increasing prevalence in the disease indicates to us that there is currently no effective control of this parasite and that in this region of Uganda at least; cysticercosis remains a neglected zoonotic disease.

## Background

In Uganda pig farming is an increasingly popular source of livelihoods [1] and the population of pigs has increased from 1.7 million in 2003 to 3.2 million 2009 [2,3]. There is a deliberate effort by the government of Uganda to encourage pig farming in poor rural communities throughPrograms like National Agricultural Advisory Services (NAADS; www.naads.or.ug). The popularity of pig farming is partially due to the ability of farmers to keep pigs under minimal input systems, allowing pigs to range freely, thereby exploiting the scavenging nature of the animals to feed them cheaply. Despite the economic opportunities which these low-input systems provide, pigs that are kept under a free range system are at high risk of acquiring a range of diseases. These diseases can either be production limiting, such as the helminths *Ascaris suis* and *Trichuris suis* [4,5] or the highly pathogenic virus causing African swine fever [6], or have serious public health consequences, such as Trichenellosis [7], Toxoplasmosis [8] and, of specific interest to this study, cysticercosis caused by the zoonotic helminth *Taenia solium* [9,10].

Cysticercus cellulosae is the larval stage of *T. solium* found in the pig. The infection is contracted by pig when they either ingest human faeces containing infective eggs, or when feeding on pasture contaminated with *T. solium* eggs [11]. In people who consume raw or inadequately cooked pork from infected animals, the larval cysts can develop into the adult stage tapeworm in their intestine, an infection known as taeniasis. Gravid proglottids containing infective eggs detach from the adult tapeworm and are excreted in the faeces in an intermittent fashion [12]. In areas where open defecation is common, the feacal material containing these infective eggs are again accessible for consumption by pigs and the life-cycle is perpetuated [13,14].

Humans can also act as an aberrant intermediate host for *T. solium* if there is faecal-oral contamination with the infective eggs. In such cases the larval stage can be found in human muscle, eye or central nervous system causing human cysticercosis [15]. The most serious form of human cysticercosis is when the larval form develops in the brain, a condition called neurocysticercosis. Neurocysticercosis is major cause of adulthood acquired epilepsy [16], making *T. solium* a great public health risk in endemic communities [17].

Key risk factors for porcine cysticercosis are present in the Lake Kyoga basin, a region of central Uganda supporting a significant rural population, as well as providing a source of food to more densely populated urban communities. The majority of pig farmers in the region keep their pigs at free range [18,19]. Open defecation is highly prevalent, with a previous study observing that 59.9% of the households do not have latrines and those with latrines often do not use them [19]. Many pigs from free-range production systems are not taken to the formal slaughter facilities where meat inspection can be carried out [20,21]. Pigs are often slaughtered in unhygienic conditions, in the open air, with slaughter men wearing no protective clothing and no cleaning of the slaughter instrument (use of one machete for slaughter, de-hairing and dressing of the carcass). Similar poor slaughter conditions have been reported in neighboring Kenya [22].

Researchers who were carrying out a study on onchocerciasis in Moyo district in northern Uganda, found that some skin nodules of human patients, suspected to be onchocerciasis were due to human cysticercosis on the skin [23]. This indicates to us that there is great need for more information about the prevalence of the disease in the pig keeping areas of the country. Lingual palpation has already been utilized to determine the prevalence of cysticercosis in this study site [19] found to range up to 12.9% in some sub-counties. Since it is known that lingual inspection is not sensitive in very light infections [24], it is possible that there is an underestimate of the true prevalence of porcine cysticercosis in the Lake Kyoga basin. A previous seroprevalence study was carried out in a smaller geographical area on the southern shores of Lake Kyoga (Kamuli and Kaliro districts) which estimated a porcine infection prevalence of 8.9% [18]. The Lake Kyoga basin region is very large, covering many districts and is currently experiencing rapid growth of the pig population. We felt there was need to carry out an extensive study on the prevalence of porcine cysticercosis in the whole of the Lake Kyoga basin. Porcine cysticercosis has a great impact on the public health of the community where it prevails [25]. The purpose of this study was to determine the sero-prevalence of porcine cysticercosis in the Lake Kyoga basin to better understand the trend of procine cysticercosis in the study areas.

## Methods

### Ethical approval

The study was carried out with authority from the Research and Ethics Committe of Makerere University Faculty of Veterinary Medicine. Approval was granted on the 14/05/2008, reference VAB/REC/08/010.

### Study area

The study area comprised those districts adjoining Lake Kyoga in central Uganda with pig populations of over 10,000 head according to the Ministry of Agriculture, Animal Industry and Fisheries [3]). Within each of these 6 selected districts, one sub-county was chosen using simple random sampling to act as the study area. The following sub-counties were sampled; Galiraya sub-county for Kayunga district, Kidera sub-county for Kamuli district, Nawaikoke sub-county for Kaliro district, Nambiesio sub-county for Apac district, Bululu sub-county for Kaberamaido district and Kangai sub-county for Amolator district. In each sub-county, the two adjoining parishes with the highest number of pigs were selected for sampling.

### Sample size determination

The sample size was determined using the formula;

$$ n= deff\frac{z^2pq}{e^2} $$ [26]

Where *deff* = design effect (2) in order to take into account the clustering associated with the design of the study which is based upon districts

*n* = sample size

*z* = the confidence interval for a normal distribution taken as 1.96 (95% level)

*p* = the prevalence of cysticercosis based on previous study in Kamuli District (p = 0.085 i[18])

*q* = 1-p

*e* = the level of precision (10%)

The minimum sample size required in each district was determined to be 60 pigs.

### Sample collection

A home-to-home-visit method was adopted to sample pigs [27]. In the study areas, most households are found along village paths. With guidance from the local authorities and the local veterinary staff, we moved along these paths from one end of the village towards the other. Houses were sampled at intervals of 400 m along the path, with the nearest pig-owning home to this distance being visited.

Piglets are unlikely to acquire *T. solium* infections before 2-6weeks [28], and *T. solium* circulating antigens are detectable 2–6 weeks post infection [29]. It was therefore decided that pigs would be eligible for inclusion in the study at three months of age. Sows in the last trimester of pregnancy were excluded for fear of causing stress resulting in abortions. One eligible pig was sampled from households with 2 of fewer pigs and 2 pigs were sampled from every homestead with 3 or more pigs with a total of 390 pig serum samples collected. The project aims and objectives were explained to pig owners and their verbal consent was obtained prior to sampling their pigs. The pig was cast in dorsal recumbencey and using a 20 gauge needle, 10 mls of blood was collected from the cranial vena cava into plain BD vaccutauners® and left at room temperature for 12 hours to allow adequate clotting. The clotted blood was centrifuged to separate the serum which was then collected and stored in cryo-vials at −20°C until laboratory analysis.

### Laboratory analysis

Samples were analyzed at the International Livestock Research Institute (ILRI) by antigen capture ELISA, utilizing the HP10 monoclonal antibody (McAb) [30]. Briefly, Flat bottom immulon® 4HB X ELISA plates were coated with 50% saturated (NH_4_) _2_S0_4_ precipitate McAb HP10 at 10 g/ml diluted in carbonate-bicarbonate buffer 9.6 pH (Sigma C3041). After being coated overnight at 4°C the plate was washed in 0.9% NaCl with 0.05% Tween®20 and non-reacted binding sites blocked for one hour at room temperature using Phosphate buffered saline (PBS) 7.3 pH (Sigma P4417) with 1% Bovine Serum Albumin (BSA) (Sigma A4503) and 0.05% Tween®20. 100 μl of undiluted sera was added to each well, incubated for one hour at 37°C, washed as before, then biotinilated McAb-HP10 diluted 1:2500 with PBS/BSA/Tween20 was added for 1 hr at 37°C. After washing again, 100 μl per well of Streptavidin peroxidise (sigma S5512) conjugate at 1:10000 dilution in PBS/BSA/Tween20 was incubated for 1 hour at 37°C, followed by a further wash before 3, 3’, 5, 5’- Tetramethylbenzidine (TMB) substrate (Sigma T8665) was added for 15 minutes at room temperature, the reaction stopped with 0.2 M H_2_SO_4_ and read in an ELISA plate reader at 450 nm. All samples were run in duplicate with five diluent control wells, five negative control wells and two positive control wells. Negative controls were obtained from indoor raised pigs from the UK which is free from *T. solium* cysticercosis and positive control sera was obtained from calves experimentally infected with *T. saginata*. Cut-off values were determined for each plate by using the mean optical density of all negative controls plus three standard deviations. Any sera sample with an optical density over this cut off was considered to be positive.

### Statistical analysis

Results were entered into Microsoft Access 2007 database and were analysed using the ‘R’ environment for statistical computing [31].

## Results

390 pigs were sampled from 6 districts in the Lake Kyoga region. The majority of pigs sampled were kept on a free-range basis although sows with suckling piglets were generally tethered.

**Figure 1:**
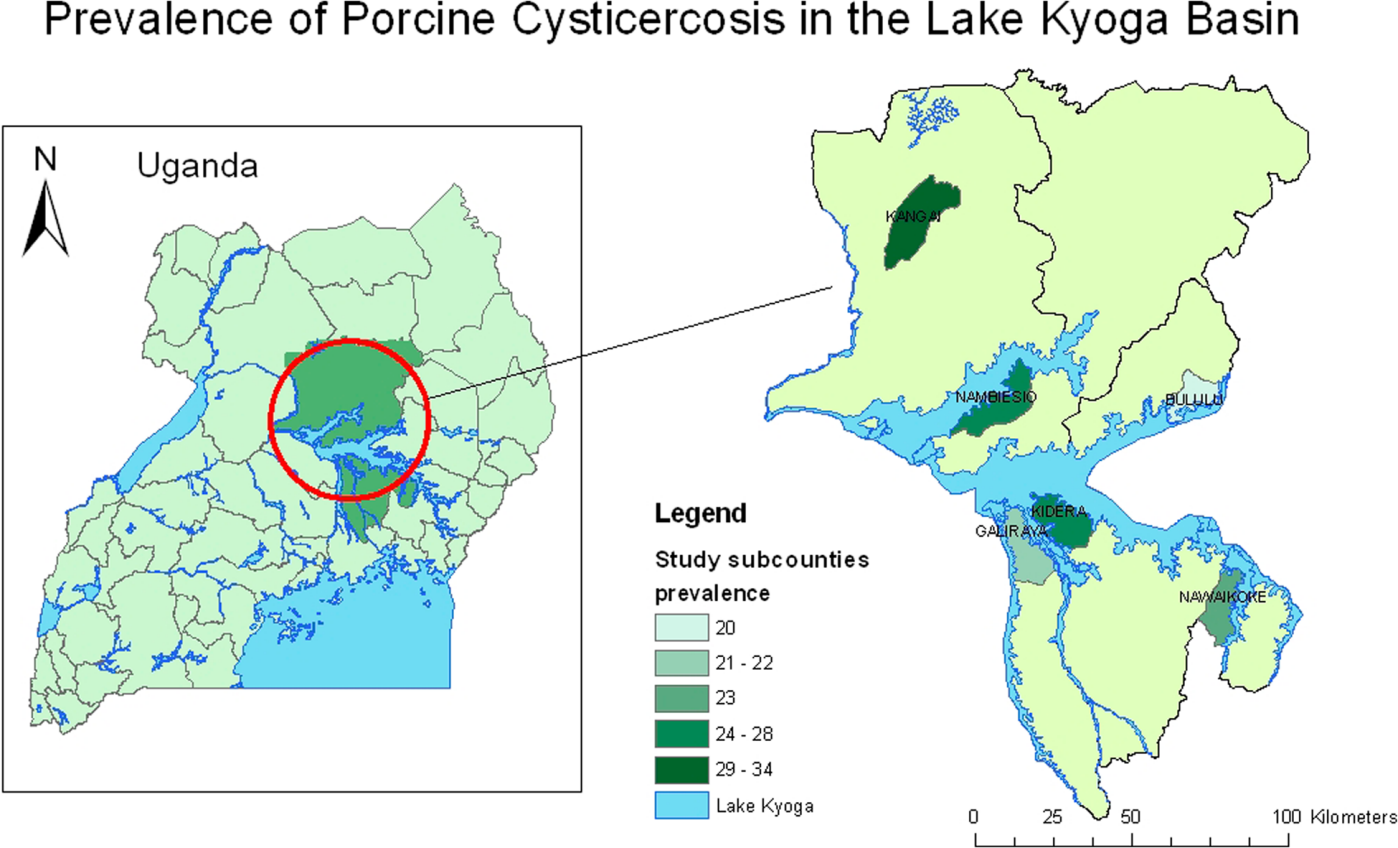
**Map of study area showing prevalence of** ***T. solium*** **cysticercosis in the porcine population of each selected sub-county.** Legend; this map was produced using ArcMap™ version9.1 with geographical data provided by ILRI GIS unit http://www.ilri.org/gis.

Sufficient sera were available to run the HP10 ELISA on 378 samples, with an average of 63 samples per district (57–69). The sero-prevalence of circulating *T. solium* antigens was found to be 25.3% (95% confidence interval 21.0-30.0%) with district prevalence ranging from 20.3-33.9% as shown in Table 1, with the geographical location of the sampling sites and their respective prevalence estimates indicated on Figure 1.

**Table 1:** **Sero-prevalence estimates and confidence intervals for each district in the study area**

**District**	**Prevalence (%)**	**95% Confidence interval (%)**
**Kayunga**	21.5	12.3-33.5
**Kamuli**	28.1	17.6-40.8
**Kaliro**	23.2	13.9-34.9
**Kiberamaido**	20.3	11.3-32.2
**Apac**	28.1	16.9-41.5
**Amolator**	33.9	22.1-47.4
**Total**	25.7	21.0-30.0

## Discussion

The prevalence of *T. solium* cysticercosis determined through the HP10 Ag-ELISA was found to be 25.3% (95% C.I. 21.0-30.0%). This is similar to the prevalence levels found in several other sub-Saharan countries where the parasite is endemic, including 34.9% prevalence in Mozambique [9], 32.8% in Kenya [32], 23.3% in Zambia [33] and 24.6% in Cameroon [34]. It is interesting to note, that a previous, smaller, study carried out in the districts of Kamuli and Kaliro in 2005 indicated a prevalence of only 8.5% (95% C.I. 6-11%) using the B60/B168 Antigen ELISA, although a rising prevalence had been noted between the 2005 study and a 2009 study using the insensitive lingual palpation method, which indicated a prevalence of up to 12.9% [19].

The drivers of increased prevalence in the study area is not clearly known, but there are several potential causes for this increase. The increasing popularity of pig keeping and pork consumption may also be one of the drivers for increased prevalence of porcine cysticercosis. An increased pig population, free ranging, in a *T. solium* contaminated environment will have higher chances to acquire infection. Although there is some evidence of successful interventions to control porcine cysticercosis in other countries [35,36], there have not been any interventions carried out to control porcine cysticercosis in the Lake Kyoga basin and the transmission cycle has therefore been allowed to continue unabated.

The main economic activity in the Lake Kyoga basin is agriculture and it is very likely that that the farmers in the field often practise open defecation due to the long distance from their hometead latrines [9]. This practice results in potentially infective feacal material being accessible by free ranging pigs, potenitally leading to increased prevalence of porcine cysticercosis.

It is also possible that this increased prevalence is due to infected pigs being moved into the study area. In Kayunga District we found that there had recently been massive deaths of pigs claimed to be due to African swine fever. The pig population was therefore comprised of new pigs acquired by farmers from outside the study area. It was therefore possible that the infected pigs acquired infection prior to moving into the study area. Unfortunately we did not obtain details of which pigs were part of this restocking procedure and this is therefore only conjecture.

The HP10 ELISA has an estimated sensitivity and specificity of 70.4% (95% Bayesian credibility interval 52.7-84.7%) ad 66.1% (B.C.I. 44.6-85.1%) respectively [37]. There is therefore the possibility that the prevalence estimate is not accurate due to undiagnosed or misdiagnosed infections, for instance due to cross reactions with *T. hydategenia* or other parasites. Fine dissection of full pig carcasses would have enabled direct visulisation of cysticercosis cellulosae and is therefore the ‘gold standard’ diagnostic for the porcine infection. Unfortunately there was no budget for such a technique and therefore the results of the angtigen ELISA provide us with the best an indication of infection prevalence.

In the absence of a detailed analysis of risk factors, the reasons for this increase in prevalence are difficult to elucidate, but we feel that the key message from this study is that no successful efforts are being made in this region to control a parasite of high public health importance. We would therefore like to strongly recommend that intervention packages for the taeniasis/cysticercosis complex, taking into account both human and porcine hosts are trialed and evaluated in order to begin the journey to eradicating this parasite.

## Conclusion

This study found a high prevalence of viable *T. solium* infection in pigs in the region of Lake Kyoga Basin. This is a serious public health risk to both the consumers of pork and the wider community and we believe that measures are required to control this important zoonotic parasite in this area.

## References

[1] Ampaire A, Rothschild MF (2010). Pigs, Goats and Chickens for Rural Development: Small Holder farmer’s Experience in Uganda. Livestock Research for Rural Development. vol. 22.

[2] MAAIF report of the pilot census of agriculture (PCA) 2003 (2004). Uganda Bureau of Statistics.

[3] The national livestock census report 20008 (2009). MAAIF/UBOS, Uganda.

[4] Stewart TB, Hale OM (1988). Losses to Internal Parasites in Swine Production. J Anim Sci.

[5] Nansen P, Roepstorff A (1999). Parasitic helminths of the pig: factors influencing transmission and infection levels. Int J Parasitol.

[6] Bengis RG, Kock RA, Fischer J (2002). Infectious animal diseases: the wildlife/livestock interface. Rev Sci Tech Off Int Epizoot.

[7] Schuppers ME, Frey CF, Gottstein B, Stärk KDC, Kihm U, Regula G (2010). Comparing the demonstration of freedom from Trichinella infection of domestic pigs by traditional and risk-based surveillance. Epidemiol Infect.

[8] van der Giessen J, Fonville M, Bouwknegt M, Langelaar M, Vollema A (2007). Seroprevalence of Trichinella spiralis and Toxoplasma gondii in pigs from different housing systems in The Netherlands. Vet Parasitol.

[9] Pondja A, Neves L, Mlangwa J, Afonso S, Fafetine J, Willingham AL, Thamsborg SM, Johansen MV (2010). Prevalence and risk factors of porcine cysticercosis in Angonia District, Mozambique. PLoS Negl Trop Dis.

[10] Sikasunge CS, Phiri IK, Phiri AM, Dorny P, Siziya S, Willingham IAL (2007). Risk factors associated with porcine cysticercosis in selected districts of Eastern and Southern provinces of Zambia. Vet Parasitol.

[11] Carrique-Mas J, Iihoshi N, Widdowson MA, Roca Y, Morales G, Quiroga J, Cejas F, Caihuara M, Ibarra R, Edelsten M (2001). An epidemiological study of Taenia solium cysticercosis in a rural population in the Bolivian Chaco. Acta Trop.

[12] Garcia HH, Gonzalez AE, Evans CAW, Gilman RH (2003). *Taenia solium* cysticercosis. Lancet.

[13] Lescano AG, Garcia HH, Gilman RH, Guezala MC, Tsang VCW, Gavidia CM, Rodriguez S, Moulton LH, Green JA, Gonzalez AE (2007). Swine cysticercosis hotspots surrounding Taenia solium tapeworm carriers. AmJTrop Med Hyg.

[14] Ngowi HA, Kassuku AA, Maeda GEM, Boa ME, Carabin H, Willingham AL (2004). Risk factors for the prevalence of porcine cysticercosis in Mbulu District, Tanzania. Vet Parasitol.

[15] Flisser A, Rodríguez-Canul R, Willingham Iii AL (2006). Control of the taeniosis/cysticercosis complex: Future developments. Vet Parasitol.

[16] Preux P-M, Druet-Cabanac M (2005). Epidemiology and aetiology of epilepsy in sub-Saharan Africa. Lancet Neurol.

[17] Boa M, Mukaratirwa S, Willingham AL, Johansen MV (2003). Regional Action Plan for Combating Taenia solium Cysticercosis/Taeniosis in Eastern and Southern Africa. Acta Trop.

[18] Waiswa C, Fèvre EM, Nsadha Z, Sikasunge CS, Willingham Iii AL (2009). Porcine cysticercosis in southeast Uganda: seroprevalence in Kamuli and Kaliro districts. J Parasitol Res.

[19] Nsadha Z, Saimo M, Waiswa C, Maingi N, Ojok L, Willingham AL, Mutagwanya R, Nyakarhuka L, Lubega G (2010). Risk factors and lingual prevalence of porcine cysticercosis in the Lake Kyoga Basin in Uganda. Africa J Anim Biomed Sci.

[20] Praet N, Kanobana K, Kabwe C, Maketa V, Lukanu P, Lutumba P, Polman K, Matondo P, Speybroeck N, Dorny P (2010). Taenia solium cysticercosis in the democratic republic of Congo: how does pork trade affect the transmission of the parasite?. PLoS Negl Trop Dis.

[21] Phiri IK, Ngowi H, Afonso S, Matenga E, Boa M, Mukaratirwa S, Githigia S, Saimo M, Sikasunge C, Maingi N (2003). The emergence of Taenia solium cysticercosis in Eastern and Southern Africa as a serious agricultural problem and public health risk. Acta Trop.

[22] Kagira JM, Maingi N, Kanyari PWN, Githigia SM, Ng‘ang’a JC, Gachohi JM (2009). Characteristics of pig trade in low income settings in Busia District, Kenya. Trop Vet.

[23] Katabarwa M, Lakwo T, Habumogisha P, Richards F, Eberhard M (2008). Could neurocysticercosis be the cause of onchocerciasis-associated epileptic seizures?. AmJTrop Med Hyg.

[24] Gonzalez AE, Cama V, Gilman RH, Tsang VCW, Pilcher JB, Chavera A, Castro M, Montenegro T, Verastegui M, Miranda E, Bazalar H (1990). Prevalence and comparision of serologic assays, necropsy, and tongue examination for the diagnosis of porcine cysticercosis in Peru. Am J Trop Med Hyg.

[25] Wandra T, Subahar R, Simanjutak GM, Margono SS, Suroso T, Okamoto M, Nakao M, Sako Y, Nakayo K, Schantz PM, Ito A (2000). Resurgence of cases of epileptic seizures and burns associated with cysticercosis in Assologaima, Jayawijaya, Irian Jaya, Indonesia, 1991–1995. Trans R Soc Trop Med Hyg.

[26] Population services international (2007). Sampling Strategies: Population Services International, Washington DC.

[27] Waiswa C, Olaho-Mukani W, Katunguka-Rwakishaya E (2003). Packed cell volume as a measure of procine health and the implication on the control of sleeping sickness in Uganda. Bulg J Vet Med.

[28] de Aluja AS, Martinez MJ, Villalobos AN (1998). *Taenia solium* cysticercosis in young pigs: age at first infection and histological characteristics. Vet Parasitol.

[29] Nguekam A, Zoli AP, Vondou L, Pouedet SM, Assana E, Dorny P, Brandt J, Losson B, Geerts S (2003). Kinetics of circulating antigens in pigs experimentally infected with T solium eggs. Vet Parasitol.

[30] Harrison LJS, Joshua GWP, Wright SH, Parkhouse RME (1989). Specific detection of circulating surface/secreted glycoproteins of viable cysticerci in Taenia saginata cysticercosis. Parasite Immunol.

[31] R Development Core Team (2005). R: A Language and Environment for Statistical Computing.

[32] Eshitera EE, Githigia SM, Kitala P, Thomas LF, Fèvre EM, Harrison LJS, Mwihia EW, Otieno RO, Ojiambo F, Maingi N (2012). Prevalence of porcine cysticercosis and associated risk factors in Homa Bay District, Kenya. BMC Vet Res.

[33] Sikasunge CS, Phiri IK, Phiri AM, Siziya S, Dorny P, Willingham AL (2008). Prevalence of *Taenia solium* porcine cysticercosis in the Eastern, Southern and Western provinces of Zambia. Vet J.

[34] Pouedet MSR, Zoli AP, Vondou L, Assana E, Speybroeck N, Berkvens D, Dorny P, Brandt J, Geerts S (2002). Epidemiological survey of swine cysticercosis in two rural communities of West-Cameroon. Vet Parasitol.

[35] Sarti E, Rajshekahar V (2003). Measures for prevention and control of Taenia solium cysticercosis in pigs in rural community in Honduras. Vet Parasitol.

[36] Ngowi HA, Carabin H, Kassuku AA, Mlozi MRS, Mlangwa JED (2008). A health-education intervention trial to reduce porcine cysticercosis in Mbulu District, Tanzania. Prev Vet Med.

[37] Krecek RC, Michael LM, Schantz PM, Ntanjana L, Smith MF, Dorny P, Harrison LJS, Grimm F, Praet N, Willingham AL (2011). Corrigendum to Prevalence of Taenia solium cysticercosis in swine from a community-based study in 21 villages of the Eastern Cape Province, South Africa. Vet Parasitol.

